# Ultrasound-based assessment of talar cartilage in individuals with chronic ankle instability: a systematic review

**DOI:** 10.1007/s10067-026-08049-3

**Published:** 2026-03-28

**Authors:** Amin Mohammadi, Saeed Eshghi, Iman Mohammadi, Patrick Wilson, Ryan McCann

**Affiliations:** 1 Ellmer College of Health Sciences, Macon & Joan Brock Virginia Health Sciences, Old Dominion University, Norfolk, VA USA; 2 Faculty of Health Sciences, Institute of Physiotherapy and Sports Science, University of Pécs, Pécs, Hungary

**Keywords:** Ankle injuries, Ankle sprain, Joint degeneration, Talar cartilage, Ultrasonography

## Abstract

**Purpose:**

To systematically synthesize ultrasound (US) evidence on talar cartilage morphology and load-induced deformation in individuals with chronic ankle instability (CAI), and to map methodological approaches (acquisition, outcome definitions, loading paradigms, and normalization) to inform protocol standardization.

**Methods:**

PubMed, Web of Science, and EBSCO were searched (March 31, 2025). Eligible studies reported quantitative US-derived talar cartilage morphology (e.g., thickness, cross-sectional area [CSA]) and/or deformation following standardized loading in CAI and comparison groups. Due to heterogeneity in imaging protocols, loading tasks, and outcome definitions, findings were synthesized narratively and organized by outcome domain.

**Results:**

Across available cohorts, load-induced deformation was the most consistently differentiating signal: CAI groups generally demonstrated greater deformation than controls, with more frequent regional effects at the medial talar dome. Evidence for baseline (unloaded) morphological differences was mixed, with some studies reporting no group differences and others reporting larger CSA in CAI. Associations between deformation and neuromechanical measures (e.g., inversion laxity, postural control, hop biomechanics, ground reaction forces) were reported but were protocol- and cohort-dependent.

**Conclusions:**

Current evidence, limited by few unique cohorts, partial non-independence across publications, and heterogeneous acquisition/loading/normalization procedures, suggests that B-mode US can quantify talar cartilage morphology and detect load-induced deformation differences in CAI in research settings. Talar cartilage ultrasonography should be considered investigational until standardized acquisition and analysis procedures and longitudinal validity are established.

**Table:** 

**Key Points** • *Ultrasound can quantify talar cartilage morphology and load-induced deformation in individuals with chronic ankle instability.* • *Load-induced talar cartilage deformation appears to be the most consistent ultrasound-derived feature differentiating chronic ankle instability from healthy controls.* • *Evidence for baseline talar cartilage morphology differences is mixed, likely reflecting heterogeneity in cohorts and imaging/loading protocols.* • *Standardized acquisition, loading, and analysis methods, along with independent longitudinal validation, are needed before clinical translation.*

## Introduction

Acute lateral ankle sprains (LAS) are among the most common musculoskeletal injuries, with ~ 2 million cases annually in the U.S. [1]. Though often considered minor, LAS can cause persistent impairments and elevate risk of ankle osteoarthritis (OA) [2, 3]. Recent evidence further demonstrates that even a single LAS can alter talar cartilage health, suggesting early degenerative changes may occur independent of recurrent injury [4]. A major complication of LAS is chronic ankle instability (CAI), a condition characterized by mechanical laxity, sensorimotor deficits, and long-term joint degeneration [5]. Individuals with CAI often present with structural changes, along with sensory-perceptual and motor-behavioral impairments [6].

Over time, repeated LAS episodes impose excessive mechanical stress on the talar cartilage, accelerating degenerative changes that may contribute to post-traumatic OA (PTOA) [7, 8]. Additionally, age-related cartilage degeneration, such as decreased proteoglycan content, exacerbates these risks by impairing the cartilage’s ability to withstand compressive forces, leading to abnormal loading patterns and microtrauma [8, 9]. Studies suggest that individuals with a history of LAS are particularly susceptible to degenerative changes in the medial talar dome, where joint stress is most concentrated [10, 11].

Magnetic resonance imaging (MRI) has strong utility for articular cartilage evaluation, as it provides a noninvasive, three-dimensional assessment, enabling both quantitative and qualitative analyses of cartilage health and disease progression, particularly in conditions like OA [12, 13]. However, its high cost and limited availability pose challenges for routine assessments in clinical practice. Ultrasonography has emerged as a promising alternative, offering a noninvasive, cost-effective, and real-time imaging modality for assessing lower extremity cartilage. A recent study reported strong correlations between ultrasound (US)- and MRI-based cartilage thickness measurements in the medial femoral condyle (*r* = 0.87–0.93), supporting the feasibility of US for cartilage evaluation [14]. Previous investigators have also reported excellent test–retest reliability for B-mode ultrasonography with a 12-MHz linear probe to assess talar cartilage thickness [15]. More recently, interrater reliability between an expert and a novice rater was also shown to be excellent across pooled B-mode US measures of talar cartilage morphology and composition obtained with a linear probe (12 MHz), with ICCs ranging from 0.92 to 0.97 [16].

While MRI studies have confirmed morphological cartilage alterations in CAI populations [17, 18], the research utility and translational potential of US for detecting talar cartilage changes, particularly under mechanical loading conditions, remain less explored. Therefore, this systematic review aimed to (1) synthesize current evidence comparing US-derived assessments of talar cartilage morphology and deformation between individuals with CAI and those without CAI, and (2) examine associations between US measures and functional outcomes, including postural control and joint mechanics. This review focuses on US assessment of the tibiotalar articular cartilage of the talar dome, imaged from the anterior ankle in plantarflexion. When reported, medial and lateral regions refer to the corresponding portions of the talar dome at the tibiotalar joint.

## Methods

This systematic review was conducted in accordance with the preferred reporting items for systematic reviews and meta-analyses (PRISMA) statement [19]. The protocol was registered on the international prospective register of systematic reviews, PROSPERO (registration: CRD420251010204).

### Eligibility criteria

Inclusion criteria were as follows:

1. Population: Individuals with chronic ankle instability (CAI).
2. Study Design: Observational studies (cross-sectional, case–control, and prospective/longitudinal) and interventional studies (e.g., randomized and nonrandomized controlled trials, or single-group pre–post designs) that report baseline quantitative talar cartilage measures.
3. Outcomes: US-based assessment of ankle articular cartilage. Primary outcomes included talar cartilage thickness, cross-sectional area (CSA), and deformation responses to mechanical loading (e.g., hopping, squatting, standing), echointensity (EI), echogenicity (EG), or other indices of cartilage health. Secondary outcomes included associations between US measurements and functional outcomes (e.g., postural control, pain, or instability measures). All US devices were eligible.

### Exclusion criteria

- Animal or cadaveric studies.
- Case reports, conference abstracts, theses, dissertations, technical reports, and review articles (systematic reviews or meta-analyses).

### Search strategy

Comprehensive literature search was conducted using the Population, Study Design, Outcomes (PSO) framework, modified to emphasize the population, comparison, and outcome components relevant to our research aim. Specifically, the search targeted studies assessing US-based evaluation of talar cartilage in individuals with CAI. PubMed, Web of Science, and the EBSCOhost research platform were searched on March 31, 2025. Within EBSCOhost, we searched all databases available through our institutional subscription at the time of the search, including key discipline-relevant databases (e.g., CINAHL and SPORTDiscus).

The search terms used included the following and were adapted for each database to account for variations in indexing:

(‘ankle instability’ OR ‘unstable ankle’ OR ‘chronic ankle instability’ OR ‘recurrent ankle sprain’ OR ‘ankle sprain*’ OR ‘ankle injur*’) AND (‘ultrasound’ OR ‘ultrasonography’ OR ‘ultrasonic’ OR ‘sonography’ OR ‘diagnostic ultrasound’) AND (‘cartilage’ OR ‘osteoarthritis’ OR ‘OA’ OR ‘echointensity’ OR ‘gray scale’ OR ‘echogenicity’ OR ‘EI’ OR ‘EG’ OR ‘cross-sectional area’).

After search results were returned, two independent reviewers (SE and IM) screened all titles, abstracts, and full texts to determine the suitability of each study for inclusion. Discrepancies were resolved through consensus with a third reviewer (AM).

### Methodological quality assessment

The methodological quality of all included studies was evaluated using the Newcastle–Ottawa Scale (NOS) adapted for cross-sectional and case–control designs [20]. Two reviewers (AM and SE) independently assessed each study across three domains: selection, comparability, and outcome/exposure assessment. Disagreements were resolved through consensus.

### Data extraction and synthesis

Two reviewers (AM and SE) independently extracted data on study characteristics (authors, year, and sample size), ultrasonography protocols, loading conditions, and main findings. Due to substantial methodological heterogeneity across the included studies, including differences in US probe frequency, imaging protocols, mechanical loading conditions (e.g., hopping vs. static stance), outcome variables (e.g., thickness, cross-sectional area, deformation percentage), and normalization approaches, a quantitative meta-analysis was not feasible. Instead, a narrative synthesis was conducted to summarize and compare trends across studies in terms of cartilage morphology and mechanical response. Where effect sizes were reported, correlation coefficients (r) were interpreted using conventional thresholds: 0.00–0.29 = negligible correlation, 0.30–0.49 = low correlation, 0.50–0.69 = moderate correlation, 0.70–0.89 = high correlation, and 0.90–1.00 = very high correlation [21]. Coefficients of determination (R2/ΔR2) were described in terms of the proportion of explained variance in cartilage deformation. Where reported, we extracted injury chronicity variables (e.g., time since initial sprain; time since most recent sprain). When injury-history timing variables were not reported, corresponding authors were contacted to obtain missing chronicity information when available.

### Equity, diversity, and inclusion (EDI) statement

Our search strategy did not exclude studies based on participants’ sex, gender, race/ethnicity, socioeconomic status, or geography. We extracted demographic data (age, sex) where available, but most included studies did not report race/ethnicity or socioeconomic context; this lack of reporting limits equity-focused interpretation and is noted as a limitation. No new participant data were collected.

Our multidisciplinary author team represents clinical and research expertise in physical therapy, athletic training, exercise science, and sports medicine. The team includes early-career, mid-career, and senior researchers, with contributions shared across conception, screening, data extraction, analysis, and drafting. This diversity of perspectives was intended to strengthen the rigor and relevance of the review.

## Results

### Study selection

**Fig. 1:**
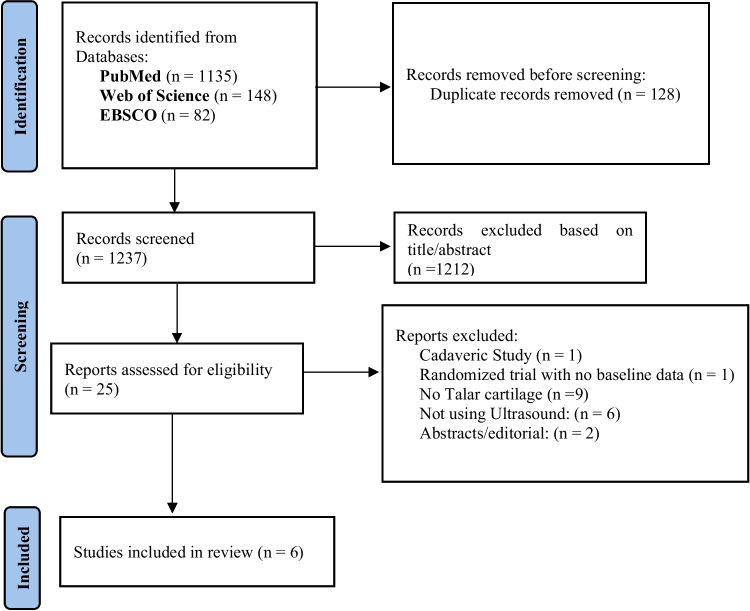
PRISMA flow diagram. PRISMA, preferred reporting items for systematic reviews and meta-analyses

The initial database search identified a total of 1365 articles (PubMed = 1135, Web of Science = 148, EBSCO = 82). After removing duplicates and screening titles and abstracts, 25 full-text articles were assessed for eligibility. Six studies met the inclusion criteria and were included in the final synthesis [15, 22–26]. Figure 1 presents the PRISMA flow diagram of the screening and selection process.

### Study characteristics

Six studies published between 2020 and 2024 met the inclusion criteria [15, 22–26]. Importantly, these six articles represent four unique participant cohorts, as three articles [22, 23, 26] analyzed the same CAI/control cohort (*n* = 60) under different loading and analytic approaches. Accordingly, similarities across these three articles should not be interpreted as independent replication. Across all included cohorts, the total number of unique participants was 198. All studies compared individuals with CAI to healthy controls, with one study [24] including an additional “coper” group (defined later). Demographic characteristics were comparable across groups in each study (Table 1).

**Table 1:** Study characteristics

Study (author, year)	Sample size	Ultrasound protocol	Loading condition	Main findings
Song et al. (2020)	15 CAI 15 Control	30-min unloading before US	N/A	No significant differences in US-derived talar cartilage thickness or normalized CSA were observed between CAI and controls at rest
Song et al. (2021)	30 CAI 30 Control	US images acquired before and after two loading protocols: static (2-min single-leg standing) and dynamic (60 single-leg hops)	Static: 2-min single-leg stance with ~ 20° knee flexion Dynamic: 60 single-leg forward hops (60 cm distance)	While resting-state talar cartilage thickness did not differ between CAI and control groups, individuals with CAI exhibited significantly greater medial and overall talar cartilage deformation than controls following both static and dynamic loading
Song et al. (2022)	30 CAI 30 Control	60-min cartilage unloading in long-sit position	60 single-leg hops (60 cm) on involved limb	Although no baseline differences were observed between groups, individuals with CAI demonstrated greater talar cartilage deformation after dynamic loading compared to controls, and this deformation was significantly associated with altered landing biomechanics and poorer functional hop test performance
Song et al. (2023)	30 CAI 30 Control	60 min of unloading in a long-sit position to allow fluid rebound	2-min static single-legged stance	Following a static loading protocol, individuals with CAI showed significantly greater overall and medial talar cartilage deformation than healthy controls, and this deformation was moderately associated with greater inversion laxity and poorer medial–lateral postural control
Kosik et al. (2022)	24 CAI 24 Control	30 min of unloading in a long-sit position	− 30 bilateral knee bends (1 min) −30 unilateral knee bends on involved limb (1 min) −10 single-limb drops from 40 cm −2-min single-leg static balance in dorsiflexion	CAI had significantly greater talar cartilage deformation than healthy controls following a standardized exercise protocol
Seo and Park (2024)	20 CAI 20 coper 20 Control	Unloading duration: 20 min in long-sit position	30-min treadmill run at RPE 13 (“somewhat hard”)	CAI had significantly greater overall, medial, and lateral talar cartilage CSA at rest compared to controls, although cartilage morphology did not change significantly after 30 min of treadmill running

*CAI* chronic ankle instability, *CSA* cross-sectional area, *US* ultrasound, *RPE* rating of perceived exertion

“Unloading” refers to non-weight-bearing rest before imaging to allow cartilage fluid recovery

“Coper” = individuals with a history of ankle sprain but without recurrent instability

Injury chronicity variables were inconsistently reported. Seo and Park reported time since the most recent lateral ankle sprain for the CAI group (28.8 ± 13.5 months) [24]. In the Song cohort, time since the initial sprain was reported (66.63 ± 44.75 months) [22, 23, 26]. For Kosik et al., time since the most recent sprain was not reported in the published manuscript but was provided by the corresponding author upon request (34.8 ± 26.3 months) [25].

### Inclusion and exclusion criteria

Five of the six included studies [15, 22, 23, 25, 26] applied inclusion and exclusion criteria consistent with the recommendations of the International Ankle Consortium (IAC) [5]. According to the IAC guidelines, CAI participants are typically identified based on the following:

- A history of at least one significant LAS,
- A minimum of two episodes of ankle “giving way” in the past six months, and
- Self-reported functional deficits as indicated by validated tools such as the Identification of Functional Ankle Instability (IdFAI) (score ≥ 11), Cumberland Ankle Instability Tool (CAIT) (score ≤ 24), or Foot and Ankle Ability Measure (FAAM) subscales.

Participants were excluded if they had recent acute lower limb injuries (within the past 3 months), history of orthopedic surgery or fractures, vestibular or visual impairments, or any neuromuscular, cardiopulmonary, or systemic conditions that could affect outcomes.

The study by [24], while not explicitly referencing the IAC, classified participants using similar criteria: at least two prior ankle sprains, CAIT ≤ 23, Foot And Ankle Ability Measure-Activities of Daily Living (FAAM-ADL) ≤ 90%, and presence of “giving way.” Their stratified approach also included coper and control groups based on symptom history and questionnaire scores. Collectively, the studies employed rigorous and comparable classification strategies to ensure diagnostic clarity and group validity.

### Quality assessment

Across studies, NOS scores ranged from 5 to 7 out of 9 stars, reflecting generally moderate methodological quality (Table 2). The most common reasons for deductions were limited control of confounders (e.g., adjustment only for weight or body mass index (BMI) without consideration of sex or age), lack of assessor blinding to case–control status, and absence of reporting on non-response rates. Only one study (Seo & Park, 2024) achieved 7/9, benefiting from blinded coded image analysis, while most others scored 6/9 due to the recurring limitations noted above. Overall, these issues suggest some risk of bias but were relatively consistent across studies.

**Table 2:** Quality assessment scores on the Newcastle–Ottawa Scale (NOS) for included studies

Study	Selection				Comparability		Exposure		
	Adequate definition of cases	Representativeness of cases	Selection of controls	Definition of controls	Comparability of cases and controls	Ascertainment of exposure	Same method of ascertainment	Non-response rate	Total stars
Song et al. (2020)	*	*	*	*	0	0	*	0	**5**
Song et al. (2021)	*	*	*	*	*	0	*	0	**6**
Song et al. (2022)	*	*	*	*	*	0	*	0	**6**
Song et al. (2023)	*	*	*	*	*	0	*	0	**6**
Kosik et al. (2022)	*	*	*	*	*	0	*	0	**6**
Seo and Park (2024)	*	*	*	*	*	*	*	0	**7**

Each asterisk (*) represents one awarded star. A maximum of 9 stars can be awarded across three domains: Selection (4 stars), Comparability (2 stars), and Exposure (3 stars). Although the table displays eight criteria, the Comparability domain can receive up to two stars, which explains why the total possible score is nine. Higher scores indicate stronger methodological quality

### Ultrasound methodologies of the included studies

All six studies utilized B-mode US to evaluate talar cartilage morphology; however, protocols varied in terms of unloading duration, participant positioning, and cartilage measurement approaches.

The unloading duration ranged from 20 to 60 min. Specifically, two studies [15, 25], implemented a 30-min unloading period, whereas other studies [22–24, 26] employed a 60-min period to allow cartilage re-expansion prior to baseline imaging.

Participant positioning was consistent, with individuals seated or supine, knees flexed to 90°, and foot placed flat on the support surface. This corresponded to approximately 140° of plantarflexion. In all studies, the transducer was placed transversely between the medial and lateral malleoli and rotated in the sagittal plane to optimize acoustic reflection from the talar articular cartilage.

Cartilage CSA was consistently calculated using ImageJ software (National Institutes of Health, Bethesda, MD), where the cartilage boundary was manually outlined with the polygon selection tool. Cross-sectional area values were normalized either to the cartilage–bone interface length [22–24, 26] or to the length of the talus in the same image [15, 25].

Four studies [22–24, 26], reported medial and lateral CSA separately, enabling regional analysis of cartilage deformation. In contrast, two studies [15, 25], presented only overall CSA or average cartilage thickness values derived from normalized CSA.

Only one included study acquired MRI and compared US measures with MRI-based talar cartilage volume [15]. They reported moderate-to-strong associations between US-derived normalized CSA and thickness with MR cartilage volume (r ≈ 0.41–0.67), supporting feasibility of the US-based talar cartilage quantification.

Observer reliability reporting was inconsistent across studies. Several investigations reported intrarater reliability for US image acquisition and/or cartilage measurement (e.g., ICCs generally ≥ 0.93), and one study [25], reported excellent intrarater segmentation reliability in a subsample. Only Seo and Park quantified both intra-rater and inter-rater reliability for talar cartilage CSA (intra-rater ICC(2,1) = 0.88–0.99; inter-rater ICC(2,k) = 0.79–0.83) [24], whereas the remaining studies primarily relied on a single experienced assessor and did not report between-rater agreement.

### Talar cartilage thickness and cross-sectional area in non-weight–bearing conditions

Only two studies directly compared talar cartilage morphology between CAI and control groups using US. One study reported no significant differences in normalized CSA or thickness between CAI and control groups following a 30-min unloading period, indicating that US did not detect structural cartilage alterations in CAI under passive, non-loaded conditions [15]. In contrast, it was reported that individuals with CAI demonstrated larger overall, medial, and lateral CSA than copers and healthy controls [24]. In this study [24], baseline morphology was assessed after a standardized unloading period prior to the running task, and we report those pre-run values as unloaded morphology, while the pre–post running comparison is summarized under loading response. In addition, within-group analyses showed no change in talar cartilage CSA across the 20-min pre-run unloading period for CAI, supporting the presence of stable baseline morphology.

### Talar cartilage deformation in response to loading

Three studies [22, 23, 26] consistently demonstrated that individuals with CAI experienced significantly greater talar cartilage deformation than healthy controls following mechanical loading. Although the consistency across these studies enhances internal validity, it is important to note that they were conducted using the same participant sample, which limits generalizability. Notably, medial cartilage deformation was consistently greater in the CAI group, particularly after static loading [23]. Another study [25] corroborated these findings by reporting a 38.4% greater reduction in normalized CSA in individuals with CAI compared to controls (–17.3% vs. –12.5%) following a standardized loading protocol including squats, balance tasks, and drop jump. The effect persisted even after adjusting for BMI, underscoring the robustness of the cartilage's vulnerability to load-induced compression in CAI populations.

One study [24] evaluated cartilage response following a treadmill running task and found no significant group × time interactions in overall, medial, or lateral CSA, indicating that deformation patterns were similar across CAI, coper, and control groups. A modest time effect was detected in the lateral cartilage, where CSA increased slightly across all groups post-run. Importantly, CAI participants consistently demonstrated larger overall, medial, and lateral CSA compared with copers and controls, reflecting baseline morphological adaptations rather than exaggerated deformation responses. Most studies suggest that individuals with CAI experience exaggerated cartilage deformation following mechanical loading, particularly in the medial dome.

### Correlational findings and predictors of cartilage deformation

One study [23] reported significant associations between cartilage deformation and neuromechanical measures in the CAI group. In the CAI group, greater inversion laxity was moderately correlated with more negative overall (r = –0.42, P = 0.03) and medial (r = –0.48, P = 0.01) talar cartilage deformation, indicating increased cartilage collapse with higher laxity. In contrast, a shorter medial–lateral time-to-boundary minima mean (reflecting poorer static balance) was moderately correlated with greater overall (r = 0.47, P = 0.01) and lateral (r = 0.50, P = 0.01) talar cartilage deformation. However, talar cartilage deformation was not associated with dynamic balance performance, measured with the star excursion balance test (SEBT). A stepwise regression model including inversion laxity and medial–lateral Time-to-boundary (TTB) explained 37% of the variance in overall talar cartilage deformation. TTB is a center-of-pressure (COP)–derived metric estimating the time required for the instantaneous COP trajectory (based on its current position and velocity) to reach the theoretical stability boundary. The mediolateral TTB minima mean reflects the mean of local minimum TTB values across a trial, with **s**maller (shorter) values indicating less time before reaching the stability limits (i.e., more frequent/closer excursions toward instability) [27].

Another study [22], reported that functional and kinetic variables significantly predicted cartilage deformation in individuals with CAI. Specifically, greater overall talar deformation was associated with longer side hop test times (ΔR2 = 0.39), higher peak vertical ground reaction force (vGRF; ΔR2 = 0.26), higher vGRF loading rate (ΔR2 = 0.23), and increased ankle plantarflexion angle at initial contact (ΔR2 = 0.35). Medial cartilage deformation was linked to reduced dorsiflexion range of motion (DFROM) (ΔR2 = 0.22), greater side hop time (ΔR2 = 0.36), higher peak vGRF (ΔR2 = 0.28), increased ankle plantarflexion angle at initial contact (ΔR2 = 0.38), and smaller peak dorsiflexion angle during stance (ΔR2 = 0.17). Similarly, lateral deformation was associated with longer side hop times, higher vGRF loading rate, and greater ankle plantarflexion at initial contact. A stepwise regression model including side hop time, ankle plantarflexion angle, and vGRF loading rate explained 76.8% of the variance in overall talar deformation in CAI participants. In contrast, the control group demonstrated only weak or inconsistent associations.

Others examined the relationship between talar cartilage deformation and basic spatiotemporal gait parameters, including gait speed, cadence, step and stride length, and the relative durations of swing, stance, single-limb support, and double-limb support [25]. After adjusting for BMI across CAI and control groups, no significant associations were observed, suggesting that these basic gait metrics may not adequately capture the mechanical vulnerability of talar cartilage.

## Discussion

This systematic review synthesized current evidence regarding talar cartilage morphology and mechanical behavior in individuals with CAI, as assessed by US under unloaded and loaded conditions. Across the included cohorts, load-induced deformation was the most consistently differentiating US-derived outcome domain, whereas baseline morphology findings were mixed. Importantly, the overall certainty of evidence is limited by a small number of unique cohorts, partial cohort non-independence, and heterogeneous acquisition, loading, and normalization procedures. Accordingly, findings should be interpreted as exploratory and most informative for protocol refinement and future validation studies.

1. Static cartilage morphology in non–weight-bearing conditions.
  Unloaded US morphology is useful for separating chronic structural characteristics of talar cartilage from its acute mechanical response to loading. Across the small and methodologically heterogeneous literature, findings for baseline (unloaded) morphology are mixed, suggesting that cohort characteristics and protocol choices can materially influence whether differences are detected between CAI and comparison groups. A plausible contributor to this heterogeneity is injury chronicity and recurrent sprain exposure, which may place participants at different points along a continuum ranging from early compositional disruption and swelling to later structural loss. However, interpretation is limited by inconsistent reporting of chronicity variables. Time since the most recent sprain was reported in two cohorts [24, 25], whereas time since the initial sprain was reported in the Song cohort [22, 23, 26]. As a result, it remains unclear whether cohorts demonstrating larger CSA reflect differences in disease duration, cumulative injury burden, or other factors. Future studies should therefore report standardized injury-history timing (time since initial sprain and time since most recent sprain) to enable stratified analyses and improve interpretability across cohorts.
  Contrary to the traditional view that cartilage degeneration leads directly to thinning, current evidence suggests that the early phase of degeneration may be marked by swelling. Studies involving the knee joint [28, 29] have shown that, in the initial stages, cartilage may undergo hypertrophy due to superficial surface damage. This damage disrupts the collagen network, reducing its ability to retain water and allowing the proteoglycan-rich matrix to absorb excess fluid, leading to transient thickening. For example, one study [29] reported that cartilage thickness either remained stable or increased in joints with early degenerative changes, with thinning observed only in more advanced stages.
  Similar findings have been reported on the ankle. Arthroscopic observations have documented swelling and surface fissures in the talar cartilage of individuals with CAI [10, 11, 30], supporting that repeated microtrauma, such as that from LAS, may initiate early degenerative processes. This may result in increased fluid content within the cartilage due to proteoglycan loss and reduced structural resistance. Consequently, the cartilage matrix expands, likely due to weakened collagen scaffolding and increased glycosaminoglycan–water interaction [31–33]. The consistent thickening observed in CAI populations across multiple studies may therefore reflect a phase of early, potentially reversible, tissue adaptation preceding irreversible structural degradation. However, longitudinal data are needed to confirm whether such thickening truly represents a transitional stage in progression or a stable adaptive response.
2. Cartilage deformation response to loading.
  Acute cartilage deformations found in the studies [22–26], suggest increased mechanical vulnerability of the talar cartilage in CAI, potentially linked to disrupted cartilage composition such as altered type II collagen and proteoglycan content [18, 34]. However, a study [24] found no significant group × time interaction in CSA following treadmill running. Despite this, the CAI group maintained consistently larger CSA values across all time points, implying chronic alterations in cartilage structure rather than acute mechanical responsiveness [24, 25]. Complementing these findings, another study observed that individuals with CAI exhibited significantly greater talar cartilage deformation in response to a standardized multi-exercise protocol that included static and dynamic weight-bearing tasks [22]. Notably, although resting-state CSA was similar between groups, the magnitude of individual cartilage deformation post-loading was higher in the CAI group, supporting the hypothesis that early cartilage changes may be compositional rather than morphological [18, 34]. These results support prior MRI-based studies demonstrating altered T1ρ and T2 relaxation times in the talar cartilage of CAI populations, indicative of early matrix degeneration [18, 34].
  While some findings [24] contradict those of the other four studies, this discrepancy likely stems from differences in task type, intensity, and biomechanical loading. Specifically, several studies [22, 23, 25, 26] employed high-impact, unilateral hopping or landing tasks that likely elevated interstitial pressure and promoted fluid exudation from the cartilage [35, 36]. In contrast, others [24] used a cyclical treadmill running protocol. This form of repetitive motion may have promoted contact migration, the movement of cartilage contact areas across the joint surface, which can facilitate fluid redistribution and mitigate localized deformation [37, 38]. Additionally, the lateral talar cartilage, which exhibited a small post-run increase in CSA regardless of group, may have been more adaptable to the dynamic inversion-eversion demands of running due to its lower stiffness and higher deformation capacity compared to the medial region [39].
  Overall, while most studies point to increased susceptibility to cartilage deformation in CAI, some findings suggest that chronic structural adaptations may mask acute mechanical responses under certain loading conditions [24]. It is also possible that the greater relative reductions reflect the thicker baseline cartilage often observed in CAI, which may amplify apparent deformation during loading.
3. Relationships between talar cartilage and neuromechanical impairments.
  Across the studies, the medial talar dome consistently exhibited greater vulnerability, manifesting either increased deformation or chronic thickening [22–24]. This region may be particularly susceptible due to its role in load bearing during the terminal stance and supination phases of gait, where inversion torques are often exaggerated in CAI. Repetitive LAS may shift joint contact pressures medially, increasing compressive loading and contributing to microstructural cartilage disruption, fluid accumulation, and early degenerative changes [30].
  A study [23] found that greater talar cartilage deformation was associated with greater inversion laxity and worse mediolateral postural control (shorter TTB values). These findings suggest that cartilage vulnerability in CAI is closely tied to joint instability and neuromechanical deficits. Importantly, static postural control was correlated with greater deformation, whereas no association was found with dynamic functional reach tasks such as the SEBT, indicating that instrumented or task-specific assessments may better capture neuromechanical contributors to cartilage loading [40, 41].
  Another study [22] reported a more complex pattern of associations. Greater talar cartilage deformation in CAI was linked to negative mechanical factors such as reduced dorsiflexion range of motion, higher ground reaction forces, and poorer hop performance, supporting the idea that altered loading mechanics can accelerate cartilage compromise [42]. At the same time, some performance measures (e.g., longer hop distances) were also associated with greater deformation, although these relationships did not remain significant in regression models. This lack of significance may reflect the limited statistical power of relatively small sample sizes (≈30 per group), in addition to the possibility that the relationship between deformation and functional performance is nonlinear and context dependent.
  In contrast, one study [25] found no significant associations between talar cartilage deformation and spatiotemporal gait parameters after adjusting for BMI. Similarly, others [22] reported that basic gait metrics did not predict deformation, despite CAI participants exhibiting greater overall deformation. These null findings may partly reflect limited statistical power given modest sample sizes, in addition to the likelihood that general gait parameters are too coarse to detect cartilage vulnerability. By contrast, standardized mechanical loading tasks appear more sensitive [43, 44].
  Overall, limited evidence currently links talar cartilage deformation to neuromechanical impairments in CAI. The associations identified in two studies [22, 23] align with the most recent CAI model [6], which posits that sensorimotor and pathomechanical factors interact to perpetuate joint compromise. However, the absence of associations in another study [25] highlights inconsistency across the literature and underscores the need for further research using standardized assessment approaches.

### Implications for research and translation

The evidence synthesized in this review reinforces CAI as a condition that can affect joint tissue health in addition to neuromuscular control. Within this framework, musculoskeletal US offers a practical approach in research settings to quantify talar cartilage morphology and acute mechanical responsiveness to controlled loading, complementing functional and biomechanical outcomes commonly targeted in rehabilitation [45, 46]. However, because current evidence is limited, talar cartilage ultrasonography should be considered investigational rather than standardized clinical assessment. Construct validity data are also limited. Only one included study directly compared US-derived talar cartilage morphology with MRI-based talar cartilage volume and reported moderate-to-strong associations between US normalized CSA/thickness and MR cartilage volume [15]. These findings support US as a feasible research tool for quantifying talar cartilage morphology. However, cross-modality agreement for load-induced deformation and responsiveness under standardized loading paradigms has not been established and remains an important area for future research.

Because musculoskeletal US is operator and analysis dependent, limited reporting of inter-observer agreement restricts inference about reproducibility across examiners and settings. Future studies should routinely report pre-specified intra and inter-rater reliability for acquisition and segmentation (plus SEM/MDC) using blind reading to support translation.

From a translational standpoint, several mechanistic lines of evidence support continued investigation of cartilage responsiveness in CAI. Cartilage composition and fluid–solid interactions can be influenced by loading history and movement behavior, and moderate physical activity has been associated with improved cartilage matrix characteristics in populations at risk of osteoarthritis [47]. In CAI, altered arthrokinematics after LAS may shift joint loading toward the medial talar dome [48, 49], providing a plausible pathway by which neuromechanical deficits and altered landing mechanics relate to regional cartilage deformation. These concepts motivate future studies that test whether interventions that modify joint loading and movement patterns also influence US-derived cartilage outcomes. However, clinical effectiveness and monitoring utility cannot be inferred from the current US literature alone. To support translation, future research should prioritize protocol standardization, including consistent ankle positioning and transducer placement and orientation, explicit medial and lateral region definitions, consistent unloading duration, reproducible segmentation workflows with reliability reporting, and harmonized normalization and deformation reporting (both absolute and percent change). Longitudinal studies are needed to determine whether load-induced deformation predicts symptoms, recurrent sprain, or osteochondral deterioration, and to establish clinically meaningful thresholds for research-to-practice translation.

### Limitations

This systematic review has several limitations. First, three included studies [22, 23, 26] used data from the same participant cohort. Although each study employed different loading protocols and outcome measures, their reliance on a single sample reduces the overall diversity and independence of the evidence base. This overlap may artificially inflate the consistency of findings and limits the generalizability of the conclusions. Therefore, the observed effects should be interpreted as exploratory rather than confirmatory. Second, the total number of studies and the total sample size were relatively small, and most participants were young, active adults, which restricts the applicability of findings to older or more sedentary populations. Additionally, because of heterogeneity in US methodologies, cartilage outcome definitions, and loading protocols, a meta-analytical synthesis was not possible. This precluded the calculation of pooled effect sizes or formal heterogeneity statistics, limiting the statistical generalizability of the findings. Moreover, construct validity against MRI was evaluated in only one included study [15], limiting inference about cross-modality agreement, particularly for deformation outcomes. Finally, while a comprehensive database search was performed, gray literature was excluded, potentially contributing to publication bias.

## Conclusion

Current evidence suggests that US can quantify talar cartilage morphology and detect load-induced deformation differences between CAI and comparison groups in research settings, with deformation outcomes showing the most consistent between-group signal. However, methodological variability limits generalizability. Standardized imaging and loading protocols and independent longitudinal validation are key next steps before clinical translation can be considered.

## Data Availability

All extracted data (study-level characteristics, outcome summaries) are available from the corresponding author upon reasonable request.
